# Undifferentiated Nasopharyngeal Carcinoma in Low- and Middle-Income Countries: Clinical, Molecular, and Health-System-Related Challenges

**DOI:** 10.7759/cureus.101207

**Published:** 2026-01-09

**Authors:** Rim Alami, Reyzane El Mjabber, Reda Alami, Asmaa Naim

**Affiliations:** 1 Radiation Oncology, Mohammed VI University of Health Sciences (UM6SS), Casablanca, MAR; 2 Radiotherapy, International University Hospital Cheikh Khalifa, Mohammed VI University of Health Sciences (UM6SS), Casablanca, MAR

**Keywords:** ebv dna, health system disparities, low and middle country (lmic), radiation and clinical oncology, undifferentiated nasopharyngeal carcinoma

## Abstract

Undifferentiated nasopharyngeal carcinoma (UNPC) is a virally driven malignancy that disproportionately impacts low- and middle-income countries (LMICs). Although advances in MRI, intensity-modulated radiotherapy (IMRT), and plasma Epstein-Barr virus (EBV) DNA testing have substantially improved outcomes in well-resourced settings, significant diagnostic and therapeutic gaps remain across endemic LMIC regions. More than 70% of global UNPC cases occur in these settings, where delayed diagnosis, limited access to MRI and PET-CT, restricted availability of EBV DNA testing, and fragile radiotherapy capacity contribute to advanced-stage presentation and poorer survival. Molecular and proteomic analyses have identified clinically relevant UNPC subgroups, but their translation into routine care is constrained by inadequate laboratory infrastructure. Systemic therapy delivery is often compromised by drug shortages, logistical challenges, and financial barriers, while substantial psychosocial distress further undermines treatment adherence. UNPC outcomes reflect an interplay between tumor biology and broader health system inequities. Strengthening referral pathways, decentralizing diagnostic services, scaling EBV DNA testing, reinforcing radiotherapy infrastructure, and integrating supportive care represent feasible, high-impact strategies to reduce survival disparities in LMICs.

## Introduction and background

Undifferentiated nasopharyngeal carcinoma (UNPC) is a virally driven tumor whose clinical behavior varies substantially across global regions. Although Epstein-Barr virus (EBV) biology drives carcinogenesis, real-world outcomes are largely determined by health system capacity. Across many low- and middle-income countries (LMICs), particularly in sub-Saharan Africa and parts of North Africa, patients continue to present with advanced-stage disease after prolonged and fragmented diagnostic pathways. These delays reflect systemic constraints, including limited access to nasopharyngoscopy, inconsistent availability of MRI, centralized pathology services, and financial barriers [1-8].

During the past decade, substantial molecular advances have clarified risk factors, epidemiology, and biological heterogeneity. Proteomic analyses from endemic regions, including North Africa, have identified clinically meaningful UNPC subgroups [9-11]. Plasma EBV DNA has emerged as a powerful biomarker for staging refinement, response monitoring, and post-treatment surveillance [11-20]. However, these advances are implemented unevenly. In many LMICs, plasma EBV DNA testing remains unavailable, endoscopy is largely confined to tertiary centers, and radiotherapy infrastructure is insufficient [8,12,13]. This Review synthesizes epidemiological, molecular, clinical, and health system evidence to characterize current gaps in care and to highlight feasible, context-appropriate interventions aimed at reducing survival disparities.

## Review

Methods

This article is a structured, non-quantitative narrative review. A focused literature search was conducted using PubMed as the primary biomedical database and was supplemented by key reports from international organizations, including the World Health Organization, the International Agency for Research on Cancer (IARC/GLOBOCAN), and the International Atomic Energy Agency. Publications published between approximately 2010 and 2025 were considered for this review. Search terms included combinations of “nasopharyngeal carcinoma,” “undifferentiated nasopharyngeal carcinoma,” “Epstein-Barr virus,” “EBV DNA,” “radiotherapy access,” “intensity-modulated radiotherapy,” “low- and middle-income countries,” and “health system disparities.” Studies were selected based on relevance to epidemiology, diagnostics, treatment delivery, health system capacity, and psychosocial aspects of UNPC in LMIC settings. Articles published in English and French were included. Evidence was synthesized using a thematic narrative approach that integrated molecular, clinical, and health system perspectives. Artificial intelligence tools were used during manuscript preparation to assist with language editing and clarity, but not for data extraction, analysis, or interpretation of the scientific content. No statistical pooling or quantitative meta-analysis was performed. No formal risk-of-bias assessment was undertaken, consistent with the narrative scope of the review.

Epidemiology and global burden

**Figure 1:**
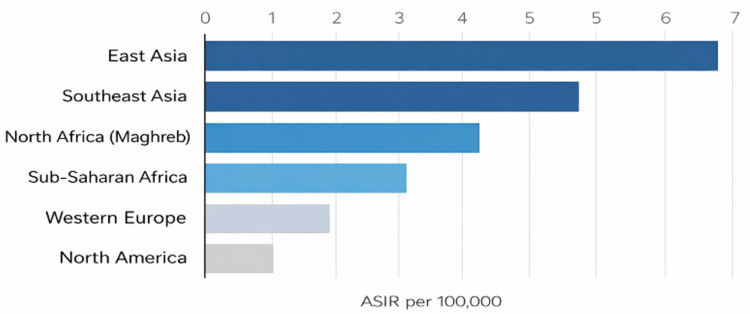
Global age-standardized incidence (ASIR) of undifferentiated nasopharyngeal carcinoma by world region Estimated incidence rates derived from published analyses based on GLOBOCAN 2020-2022 data [6]. Incidence is highest in East and Southeast Asia, intermediate in North Africa and sub-Saharan Africa, and lowest in Western Europe and North America Image credit: Alami Rim

UNPC exhibits a highly uneven global distribution that is driven by viral, environmental, and systemic determinants. An estimated 129,000 new cases occur annually, with the majority occurring in LMICs, where risk factors such as early-life EBV exposure, nitrosamine-rich preserved foods, and indoor air pollution remain common [1-3]. Age-standardized incidence rates reach 8-15 per 100,000 in endemic regions of East and Southeast Asia and the Maghreb, compared with fewer than 1 per 100,000 in most Western countries [3-5]. Regional variations in incidence are illustrated in Figure 1.

A consistent male predominance (2:1 to 3:1) and a trend toward older age groups have been observed [4-6]. More than 60% of patients in LMICs present with stage III-IV disease [6-8], particularly in sub-Saharan Africa and parts of North Africa, which reflects diagnostic delays, limited specialist availability, and restricted access to imaging. UNPC accounts for over 80,000 deaths annually [3]. The true burden is likely underestimated because many LMICs lack population-based cancer registries [7]. This lack of surveillance systems complicates policymaking and hinders targeted investment in diagnostic and treatment capacity. The global availability of population-based cancer registries is shown in Table 1.

**Table 1:** Population coverage of population-based cancer registries by region Coverage refers to the proportion of the population included in population-based cancer registries. Categories (robust >80%, moderate 20-80%, limited <20%) represent an author-defined classification, derived from international cancer surveillance reports (IARC) to facilitate comparative analysis across regions [8] Table credit: Alami Rim

Region	Population covered by high-quality cancer registries	Classification
North America	>95%	Robust
Western Europe	80–100%	Robust
Australia/NZ	>95%	Robust
East Asia	40–70%	Moderate
Southeast Asia	10–40%	Limited
Maghreb/North Africa	5–20%	Limited
Latin America	8–50%	Limited-moderate
Sub-Saharan Africa	<10%	Very limited
Middle East	20–60%	Moderate

The persistence of these gradients underscores that UNPC outcomes reflect structural inequities. While EBV infection is ubiquitous, differences in referral pathways, imaging availability, radiotherapy capacity, and biomarker access drive survival disparities

Etiology and pathogenesis

UNPC is an EBV-associated malignancy influenced by viral latency programs, host susceptibility, and environmental co-factors. EBV proteins (LMP1, LMP2A, EBNA1) activate NF-κB, PI3K-AKT, and JAK-STAT pathways, thereby promoting proliferation, immune escape, and treatment resistance. Geographic variation in EBV strains (such as China 1 versus the prototype B95-8 strain) has been suggested as a factor contributing to regional differences in UCNT incidence and biology, although its clinical significance remains uncertain. Epigenetic dysregulation and genomic instability also play a role in oncogenesis [9-11].

Environmental exposures substantially increase risk in endemic regions. These include early-life EBV infection, nitrosamine-rich preserved foods, indoor air pollution, and household biomass combustion, all of which remain common in many areas [4,5]. Host genetic factors, including specific HLA haplotypes, further influence susceptibility [4]. Proteomic studies from Morocco and Southeast Asia have identified tumor clusters characterized by immune signatures and pathway activation (PI3K, MAPK, BCL2) [9-14]. Although these findings demonstrate biological diversity within UCNT, molecular profiling rarely informs clinical practice in LMICs due to limited laboratory infrastructure. Overall, UNPC pathogenesis reflects a complex interaction between viral carcinogenesis, environmental exposures, and host factors, with molecular insights increasingly identifying potential targets for risk stratification and future therapeutic strategies.

Plasma EBV DNA as a biomarker

Plasma EBV DNA is one of the most powerful prognostic biomarkers in UNPC. High pre-treatment levels more than double the risk of mortality, while persistent post-treatment positivity increases the risk of recurrence and metastasis by four- to eightfold [13-20]. In high-income countries, plasma EBV DNA is used to guide staging, response assessment, therapeutic decisions, and follow-up. Patients with undetectable post-treatment plasma EBV DNA achieve three-year overall survival above 85%, whereas persistently elevated plasma EBV DNA is associated with poorer outcomes, including recurrence or metastasis. Clinical guidelines also recognize plasma EBV DNA as a valuable complement to TNM staging, allowing for more accurate risk stratification [13-23].

Moroccan studies indicate that more than 90% of UNPC patients have detectable plasma EBV DNA at diagnosis, with higher levels linked to advanced stage, nodal burden, and metastasis [21-27]. Longitudinal cohorts confirm that persistent plasma EBV DNA at 6-24 months predicts inferior progression-free survival [24-29]. Early research demonstrated that EBV DNA more accurately reflects tumor burden than serological markers such as VCA/IgA [30-31]. Despite its utility, plasma EBV DNA testing remains limited in many LMICs due to cost, centralized laboratory requirements, and logistical barriers.

Clinical presentation and diagnostic delay

In endemic LMICs, UNPC often resembles benign ENT conditions, contributing to delayed recognition. Common presenting symptoms include cervical lymphadenopathy, nasal obstruction, epistaxis, and serous otitis media [9-10]. Because initial presentation frequently occurs in primary care settings, limited training of non-specialist providers, including general practitioners and nurses, in recognizing UNPC red-flag symptoms remains a critical barrier to timely referral. Targeted training programs and simplified referral criteria for frontline providers could substantially reduce diagnostic intervals in resource-constrained settings. Median symptom-to-diagnosis delays of six to eight months are widely reported across LMICs, especially in North Africa and Southeast Asia [32-36]. Each additional month increases the risk of stage progression and lowers survival [18,36-39].

MRI is preferred for locoregional staging due to its superior soft-tissue resolution [19,21]. However, MRI availability is limited and often centralized in urban tertiary centers. PET-CT is recommended for detecting distant metastases but remains inaccessible in large parts of LMICs, especially sub-Saharan Africa and the Maghreb, because of cost, radiotracer shortages, and machine downtime [19,22-25]. Consequently, many LMICs rely on CT, bone scintigraphy, and ultrasound modalities with lower diagnostic accuracy, increasing the risk of understaging. Plasma EBV DNA complements imaging for prognostic assessment and post-treatment surveillance [13-17,27]. However, uptake is limited due to laboratory constraints and a lack of standardized protocols. Children experience even longer delays. In a Moroccan series, the mean time to diagnosis was 4.5 months, with lymphadenopathy present in 90% of cases [27]. This underscores gaps in pediatric ENT pathways and community awareness. As shown in Table 2, diagnostic capacity differs widely across regions.

**Table 2:** Global disparities in diagnostic access relevant to nasopharyngeal carcinoma This table summarizes regional differences in access to core diagnostic modalities required for the timely evaluation of undifferentiated nasopharyngeal carcinoma, including nasopharyngoscopy, MRI, pathology services, and EBV DNA testing. Access levels are categorized as robust, moderate, or limited based on published analyses of health-system capacity in low-, middle-, and high-income countries. Sources: WHO 2021; IAEA 2022 [40-42] MRI: magnetic resonance imaging; PET-CT: positron emission tomography/computed tomography; EBV: Epstein-Barr virus; WHO: World Health Organization; IAEA: International Atomic Energy Agency Table credit: Alami Rim

Modality	MRI availability (per million population)	PET-CT availability	ENT specialists density (per 100,000)	Plasma EBV DNA testing availability
Region				
East Asia	6–12	High	1.5–3.0	Widely available
Southeast Asia	1–4	Moderate	0.5–1.5	Available in tertiary centers
North Africa (Maghreb)	1–2	Limited	0.4–0.8	Limited/regional hubs
Sub-Saharan Africa	<1	Very limited	<0.2	Rare/unavailable
Western Europe	10–40	High	2–4	Widely available
North America	30–40	High	3–4	Widely available

Treatment strategies

Radiotherapy is the primary curative treatment for UNPC, with intensity-modulated radiotherapy (IMRT) providing excellent local control when delivered without interruptions [21,22,35]. However, access to radiotherapy represents one of the most significant inequities in global oncology. According to the International Atomic Energy Agency Directory of Radiotherapy Centers (IAEA DIRAC), approximately 10,500 machines serve over 190 countries, but their distribution is highly uneven [23,24]. Several LMICs, including many in sub-Saharan Africa and the Maghreb, have fewer than one machine per million inhabitants, far below the recommended minimum of four [23-25]. Global radiotherapy distribution is illustrated in Table 2, based on the IAEA DIRAC database, regional summaries from IAEA technical reports, and published global oncology disparity reviews [23].

**Table 3:** Global radiotherapy availability and density of IMRT-capable machines ^*^Machine density is expressed per million population and includes LINACs and cobalt units. Adequate capacity corresponds to ≥5 machines per million, limited to 1-4 machines per million, and scarce to <1 per million [23] IMRT: intensity modulated radiotherapy; LINAC: linear accelerator Table credit: Alami Rim

Region	Radiotherapy machine density (per million population)*	Infrastructure classification	Notes
North America	12–15	Adequate	High LINAC availability, stable maintenance programs
Western Europe	8–12	Adequate	Predominantly LINAC-based, strong physicist workforce
East Asia	3–6	Limited-adequate	Large variability: Japan high, China moderate, Mongolia low
Southeast Asia	1–3	Limited	Heavy reliance on public-sector LINACs; maintenance interruptions are common
North Africa (Maghreb)	0.8–1.5	Limited	Morocco/Tunisia moderate; others lower
Latin America	1–2	Limited	Urban-rural disparity is significant
Sub-Saharan Africa	<0.5	Scarce	Many countries have no radiotherapy at all
Oceania (high-income)	9–12	Adequate	Australia/NZ is well-equipped
Middle East	1–4	Limited	Strong centres in Gulf states; scarcity elsewhere

Frequent machine breakdowns, limited maintenance programs, and shortages of medical physicists contribute to prolonged waiting lists and unplanned treatment gaps, all of which are known to compromise tumor control. In some LMIC centers, the interval between LINAC failures is up to three times shorter than in high-income settings, substantially affecting treatment adherence. Even when radiotherapy is available, limited image guidance or adaptive planning can lead to suboptimal dosing and increased toxicity. Concurrent chemoradiotherapy (CCRT) with high-dose cisplatin remains the standard of care for locoregionally advanced UNPC [22]. Meta-analyses confirm that CCRT reduces mortality by approximately one-third, while induction chemotherapy (ICT) enhances distant control in bulky disease [21].

In many LMICs, systemic therapy delivery is often hindered by drug shortages, inadequate antiemetic support, limited infusion capacity, and financial constraints [26]. These limitations frequently result in incomplete cycles or dose reductions, diminishing the effectiveness of otherwise standard regimens. Immunotherapy represents a promising option for recurrent or metastatic UNPC, but access remains extremely limited in LMICs due to cost, regulatory delays, and the scarcity of clinical trials [26]. Similarly, EBV DNA-guided treatment adaptation, widely implemented in well-resourced settings, is not possible without consistent biomarker availability [19].

Psychosocial burden

UNPC imposes a substantial psychosocial burden, particularly in LMICs, where younger adults often constitute a large proportion of patients. Rates of clinically significant anxiety, depression, and distress exceed 30-40% during chemoradiotherapy [36-39,43]. Sleep disturbances and mood symptoms are frequently reported [37]. Stigma, travel distance, and financial toxicity all markedly affect treatment adherence. Many patients must relocate or travel long distances for radiotherapy, incurring costs for transportation, accommodation, and lost income. These pressures contribute to treatment abandonment, a well-documented driver of poor outcomes in LMICs. Rehabilitation services for xerostomia, dysphagia, and hearing loss, which are common late toxicities, are often limited, worsening long-term functional impairment [36-39]. Evidence suggests that community health worker support, peer programs, and telepsychology can alleviate distress and enhance adherence, yet these resources remain scarce [39].

Health disparities and systemic drivers

Global outcome disparities in UNPC primarily reflect differences in diagnostic capacity, radiotherapy infrastructure, systemic therapy delivery, and psychosocial support, rather than being driven by biological heterogeneity alone. Importantly, LMICs are not a homogeneous group, and substantial intra- and inter-country variation exists. Access to radiotherapy and advanced diagnostics is often higher in upper-middle-income or urban settings, such as parts of North Africa, than in low-income or rural regions, particularly in sub-Saharan Africa [24,25].

These structural constraints are most pronounced in sub-Saharan Africa, where published series from countries including Nigeria and Kenya report predominantly advanced-stage presentation and extremely limited radiotherapy availability, often below 0.5 machines per million population, highlighting the severity of systemic gaps in the region. Fragmented referral pathways, limited access to nasopharyngoscopy, and centralized imaging further delay diagnosis, while financial barriers impede continuity of care [31,37,40-42]. Conversely, health systems with integrated referral pathways, broader diagnostic access, and reliable radiotherapy infrastructure are associated with improved cancer outcomes, even without widespread access to advanced molecular tools. From a resource-allocation perspective, EBV DNA-guided monitoring may further support cost-efficient care by improving clinical triage and reducing the need for repeated imaging, thereby optimizing the use of constrained diagnostic resources. A structured comparison of health-system capacities relevant to UNPC is summarized in Table 4.

**Table 4:** Comparative health-system capacities relevant to UNPC care in LMICs and high-income countries This table contrasts key determinants of undifferentiated nasopharyngeal carcinoma outcomes across settings, including diagnostic access, radiotherapy infrastructure, systemic therapy availability, psychosocial support, and data systems [23,31,37,40-42] UNPC: undifferentiated nasopharyngeal carcinoma; LMICs: low- and middle-income countries; MRI: magnetic resonance imaging; PET-CT: positron emission tomography/computed tomography; EBV: Epstein-Barr virus; IMRT: intensity modulated radiotherapy Table Credit: Alami Rim

Domain	LMICs	High-income countries	Impact on outcomes
Diagnostic access	Limited nasopharyngoscopy, delayed MRI/PET-CT use; plasma EBV DNA testing scarce or centralized	Ready access to nasopharyngoscopy, MRI, PET-CT, and routine plasma EBV DNA	Diagnostic delays; stage migration; poorer prognostication
Radiotherapy capacity	<1 machine per million population; frequent equipment downtime; limited IMRT access	≥4 machines per million; widespread IMRT	Suboptimal dosing; higher toxicity; compromised local control
Systemic therapy access	Cisplatin chemoradiotherapy variable; induction and immunotherapies are limited by infrastructure and cost	Standardized full chemoradiotherapy; immunotherapy trials and approvals available	Incomplete systemic therapy limits control of bulky and metastatic disease
Psychosocial support	Sparse psychological services; stigma; financial toxicity high	Structured psycho-oncology; patient navigators	Non-adherence; treatment abandonment; poorer quality of life
Data infrastructure	Limited cancer registries; sparse quality improvement data	Robust registries; electronic health record integration	Difficult to measure outcomes and tailor interventions

Recommendations

Reducing UNPC disparities will require coordinated action at multiple levels of the health system. Although some interventions are resource-intensive, many are practical and achievable in LMIC settings. High-impact, short-term priorities include strengthening early detection and referral pathways and improving access to essential diagnostic tools, while longer-term investments are needed to expand radiotherapy capacity and maintain advanced technologies. Implementation of these strategies is most effective when integrated with national cancer control plans and supported by appropriate health financing mechanisms.

Early detection needs significant improvement. In many LMICs, the pathway from symptom onset to ENT evaluation remains unpredictable [33]. Standardized referral criteria for primary care providers, community awareness campaigns, and simple electronic referral-tracking tools could substantially shorten time to diagnosis. Diagnostic infrastructure also requires expansion. Nasopharyngoscopy and MRI, which are cornerstones of UNPC evaluation, remain concentrated in tertiary centers. Establishing satellite ENT clinics, implementing tele-endoscopy, and developing regional MRI hubs could broaden access [8,19] and reduce delays.

Plasma EBV DNA testing should be scaled thoughtfully. Although nationwide implementation may be challenging, regional plasma EBV DNA hubs with reliable sample transport are practical, cost-effective, and provide immediate benefits. In resource-constrained settings, EBV DNA-guided surveillance may also enhance cost-effectiveness by improving risk stratification and reducing unnecessary imaging and low-yield follow-up in selected patients. Evidence from Moroccan and Asian cohorts [27-29] demonstrates that integrating plasma EBV DNA improves prognostic accuracy and surveillance.

Complementary system-level measures, such as establishing reliable sample transport networks for plasma EBV DNA testing and exploring AI-assisted imaging interpretation in settings with limited specialist availability, may further strengthen diagnostic capacity and efficiency in resource-limited environments. Radiotherapy access must be expanded in both availability and reliability. Investment in IMRT-capable machines, preventive maintenance, and training programs for medical physicists and radiation oncologists would stabilize radiotherapy delivery, a critical issue for radiosensitive tumors such as UCNT [21,24]. Systemic therapy delivery also requires operational strengthening. Drug shortages, limited hydration support, and restricted infusion capacity contribute to incomplete chemoradiotherapy [22,23]. Ensuring chemotherapy availability and expanding day-hospital infusion capacity would minimize treatment interruptions.

Psychosocial care should be formally integrated. Distress is common in UNPC, and even modest interventions, such as telepsychology, financial navigation services, or support from community health workers, can improve adherence and quality of life [36-39,43]. Cancer registry coverage must be expanded. The absence of robust registries in many LMICs impedes planning for radiotherapy infrastructure, workforce training, and diagnostic capacity [7]. Reliable data are essential for long-term cancer-control strategies. Taken together, these recommendations represent realistic, high-yield interventions capable of meaningfully improving outcomes in resource-limited settings.

Limitations

This review has several limitations. Data quality and availability across LMICs remain uneven, and many regions lack population-based cancer registries, making accurate estimation of incidence and survival challenging. Much of the available evidence comes from tertiary referral centers, which may not represent broader community-level patterns. Additionally, several proposed interventions, particularly those involving molecular tools, have not undergone prospective validation in LMIC settings. Finally, although efforts were made to include non-English literature when accessible, regional publications remain incompletely represented.

## Conclusions

UNPC lies at the intersection of viral carcinogenesis and structural inequity. While advances in molecular characterization, plasma EBV DNA monitoring, and radiotherapy have improved outcomes in high-income countries, these benefits remain largely inaccessible in many LMICs, perpetuating a gap between scientific progress and real-world care. Reducing this disparity will require pragmatic reforms, strengthened referral pathways, decentralized diagnostics, and reliable radiotherapy capacity, underpinned by sustained health-system investment. Coordinated action by national ministries of health, global funding bodies, and cancer alliances is essential to translate evidence-based strategies into practice and bring the benefits of contemporary UNPC management to the populations most affected by the disease.
